# Supplementary material for: Deciphering Structural Determinants in Chondroitin Sulfate Binding to FGF-2: Paving the Way to Enhanced Predictability of Their Biological Functions
Source: Polymers (Basel). 2021 Jan 19;13(2):313. doi: 10.3390/polym13020313 (PMC7835997; doi:10.3390/polym13020313)
Supplement: Supplementary file 1 [file polymers-13-00313-s001.pdf]

polymers

Supporting Information

Deciphering Structural Determinants in Chondroitin Sulfate Binding to FGF-2: Paving the Way to Enhanced Predictability of their Biological Functions

Giulia Vessella [1], José Antonio Vázquez [2], Jesús Valcárcel [2], Laura Lagartera [3], Dianélis T. Monterrey [4], Agatha Bastida [4], Eduardo García-Junceda [4], Emiliano Bedini [1], Alfonso Fernández-Mayoralas [4] and Julia Revuelta [4,*]

[1] Department of Chemical Sciences University of Naples Federico II, Via Cinthia 4, I-80126 Naples, Italy; giulia.vessella@unina.it (G.V.); ebedini@unina.it (E.B.)
[2] Group of Recycling and Valorization of Waste Materials (REVAL), Marine Research Institute (IIM-CSIC), Eduardo Cabello, 6, 36208 Vigo, Galicia, Spain; jvazquez@iim.csic.es (J.A.V.); jvalcarcel@iim.csic.es (J.V.)
[3] Institute of Medicinal Chemistry (CSIC), Juan de la Cierva 3, 28006 Madrid, Spain; l.lagatera@iqm.csic.es
[4] BioGlycoChem Group. Institute of General Organic Chemistry (CSIC), Juan de la Cierva 3, 28006 Madrid, Spain; d.monterrey@iqog.csic.es (D.T.M); agatha.bastida@csic.es (A.B.); eduardo.junceda@csic.es (E.G.-J.); alfonso.mayoralas@csic.es (A.F.-M.)
* Correspondence: julia.revuelta@iqog.csic.es; Tel.: +34-(91) 2587679

Citation: Vessella, G.; Vázquez, J.A.; Valcárcel, J.; Lagartera, L.; Monterray, D.T.; Bastida, A; García-Junceda, E.; Bedini, E.; Fernández-Mayoralas, A.; Revuleta, J. Deciphering Structural Determinants in Chondroitin Sulfate Binding to FGF-2: Paving the Way to Enhanced Predictability of their Biological Functions. *Polymers* **2021**, *13*, 313. https://doi.org/10.3390/polym13020313

Academic Editor: Jalel Labidi

Received: 29 December 2020
Accepted: 15 January 2021
Published: 19 January 2021

Publisher's Note: MDPI stays neutral with regard to jurisdictional claims in published maps and institutional affiliations.

Copyright: © 2021 by the authors. Licensee MDPI, Basel, Switzerland. This article is an open access article distributed under the terms and conditions of the Creative Commons Attribution (CC BY) license (http://creativecommons.org/licenses/by/4.0/).

1. Synthesis of CS-9, CS-10, CS-13, CS-14, and CS-15

1.1. Synthesis of CS-9

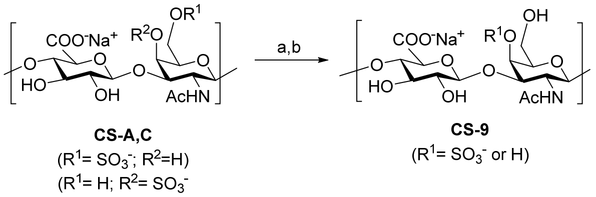

(a) (i) IRA-120 (H^+ form), H_2O, (ii) *n*-Bu_3N, r.t., 30 min; (b) MSTFA, Py, 100 °C, 20 h.

CS-9 was prepared from Bu_3N·**CS-6** salt. This salt (150 mg) was dissolved in pyridine (15 mL) and *N*-methyl-*N*-trimethylsilyltrifluoroacetamide (0.37 mL) was added. The reaction mixture was stirred at 100 °C for 20 h, and later this was cooled at 0 °C and water (3 mL) was added, obtaining a solution that was dialyzed against Milli-Q water for 18 h. The new solution was acidified by addition of Amberlite® IR-120(H^+ form) (500 mg), being removed the resin after 15 min of stirring at room temperature by filtration. Finally, the pH of the obtained solution was increased until pH 10.0 with NaOH (aq) (2 M), and the mixture was stirred for 2 additional hours. Finally, this was dialyzed against deionized water using a 3.5 kDa cut-off dialysis membrane for 24 h and freeze-dried to obtain **CS-9** (yield: 72%).

1.2. Synthesis of CS-10

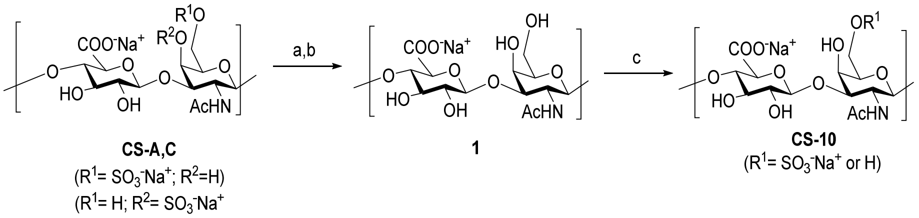

(a) (i) IRA-120 (H^+ form), H_2O, (ii) *py*, r.t., 30 min; (b) DMSO/H_2O (10:1), 100 °C, 7 h; (c) SO_3·py, DMF, 80 °C, 5.5 h.

CS-10 was prepared from py·**CS-6** salt. Firstly, this salt was totally desulfated following the conditions described by Nagasawa et al [1]. In a second step, the obtained desulfated-CS was regioselectively 6-*O*-sulfated. To do this, the polysaccharide (50 mg) was dissolved in dry DMF (4 mL) and SO_3·py (49 mg) was added. The obtained mixture was stirred at 80 °C for 5.5 h and then the pH was increased until pH 10.5 with NaOH (aq) (2 M), and the mixture was stirred for 2 additional hours. The obtained solution was dialyzed against deionized water using a 3.5 kDa cut-off dialysis membrane and freeze-dried to obtain **CS-10** (yield: 62%).

1.3. Synthesis of CS-13, CS-14 and CS-15

1.3.1. General Procedure for Sulfation and Global Deprotection.

Intermediate polysaccharide (see below) (40 mg) was dissolved in DMF (1.0 mL), which was freshly dried over 4 Å molecular sieves and then treated with a 0.90 M solution of SO_3·py complex in freshly dried DMF (1.7 mL). After overnight of stirring at 50 °C, a saturated NaCl solution in acetone (4 mL) was added at r.t. The obtained yellowish precipitate was collected by centrifugation and then suspended in deionized water (5.0 mL). The acid mixture (pH 2) was heated to 50 °C and stirred for 2 h to give a yellowish solution that was cooled to r.t. and then treated with a 4 M NaOH solution to adjust pH to 12. The solution was stirred at r.t. overnight, and then 1 M HCl was added until neutralization. Dialysis and subsequent freeze-drying yielded final polysaccharide. To obtain **CS-13** an additional alkaline hydrolytic reaction was performed by treating a solution of the obtained solid (23 mg in 1.9 mL of H_2O) first with 2:1 v/v aqueous 1 M LiOH-30% H_2O_2 (1.4 mL, pH 10) at r.t. overnight and then with 4 M NaOH solution to adjust pH to 12. The obtained solution was stirred at r.t. overnight, then neutralized with 1 M HCl, and finally, treated with 1M NaCl (2.9 mL). After 1 h of stirring at r.t., the solution was dialyzed and subsequently freeze-dried to yield final polysaccharides.

1.3.2. Synthesis of CS-13

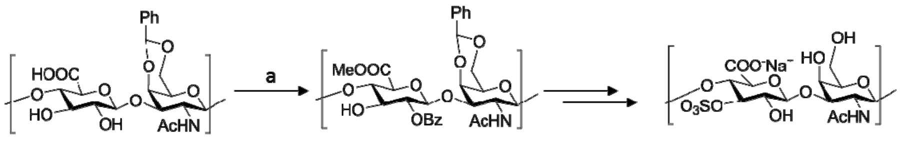

2 **3** **CS-13**

(a) (i) Bz_2O, DMF, 85 °C, 26 h; (ii) DMAP, py, r.t., 68 h; (iii) AcONa, MeOH, r.t., 26 h.

CS-13 was prepared from polysaccharide **2** which was obtained in turn following the conditions described by us [2]. This intermediate **2** (114 mg, 0.242 mmol repeating unit) was dissolved under an Ar atmosphere in DMF (5.0 mL), which was freshly dried over 4 Å molecular sieves. The solution was treated with Bz_2O (1.64 g, 7.26 mmol) and then heated to 85 °C. After 26 h of stirring, it was cooled to r.t. and treated with pyridine (4.3 mL) and DMAP (59.1 mg, 0.484 mmol). The yellowish solution was stirred at r.t. for 68 h and then treated with methanol (4.3 mL) and sodium acetate (29.8 mg, 0.363 mmol). After 26 h of stirring, it was concentrated by rotoevaporation to approximately 10 mL in volume and then treated with diisopropyl-ether (25 mL). The obtained white precipitate was collected by centrifugation, then dissolved in DMSO (4 mL), and precipitated again with 2:1 v/v acetone-diisopropyl ether (18 mL). The white solid was collected by centrifugation and dried under vacuum overnight to give crude derivative **3**. Subsequently, this intermediate was sulfated and deprotected according with general procedures described above (section 1.3.1).

1.3.3. Synthesis of CS-14.

CS-14 was obtained by direct sulfation of polysaccharide **2**, followed by a global deprotection (see section 1.3.1).

1.3.4. Synthesis of CS-15.

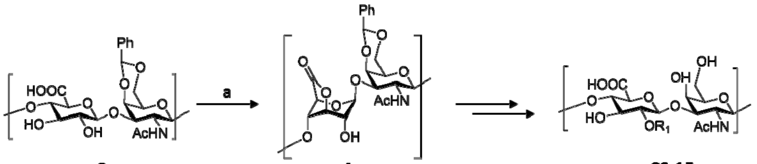

2 **4** **CS-15**
R_1= $SO_3^-Na^+$ or H

(a) Bz_2O, DMF, 85 °C, 26 h.

CS-15 was prepared form polysaccharide **2** which was obtained in turn following the conditions described by us [2]. This polysaccharide **2** (44.4 mg, 94.7 μmol repeating unit) was dissolved under an Ar atmosphere in DMF (2.0 mL), which was freshly dried over 4 Å molecular sieves. The solution was treated with Bz_2O (642 mg, 2.84 mmol) and then heated to 85 °C. After 26 h of stirring, it was cooled to r.t. and treated with diisopropyl-ether (12 mL). The obtained white precipitate was collected by centrifugation and dried under vacuum overnight. Crude derivative **4** (39.2 mg, 88.3% weight yield) was obtained as a white powder. Subsequently, this intermediate was sulfated and deprotected according with general procedures described above (section 1.3.1).

2. Supplementary Tables

Table S1. ^{1}H (plain) ^{13}C (italic) NMR chemical shift attribution of **CS-7**.

<table>
<tr><th>Residue</th><th>1</th><th>2</th><th>3</th><th>4</th><th>5</th><th>6</th><th>Other Signals</th></tr>
<tr><td>GlcA</td><td>4.47
105.1</td><td>3.37
73.5</td><td>3.60
75.1</td><td>3.77
83.5</td><td>3.69
77.8</td><td>---</td><td></td></tr>
<tr><td>GalNAc4,6S</td><td>4.61
102.8</td><td rowspan="2">4.04
52.6</td><td>4.04
76.8</td><td>4.79
77.4</td><td>4.11
73.7</td><td>4.24
68.9</td><td rowspan="2">NAc: 2.02/23.8</td></tr>
<tr><td>GalNAc</td><td>n.d</td><td>n.d.</td><td>n.d.</td><td>n.d.</td><td>3.77
62.3</td></tr>
</table>

Table S1. ^{1}H (plain) ^{13}C (italic) NMR chemical shift attribution of **CS-8**.

<table>
<tr><th>Residue</th><th>1</th><th>2</th><th>3</th><th>4</th><th>5</th><th>6</th><th>Other Signals</th></tr>
<tr><td>GlcA2S</td><td>4.70
103.2</td><td>4.11
80.7</td><td>3.80
74.1</td><td>3.89
81.1</td><td>3.73
77.9</td><td>---</td><td></td></tr>
<tr><td>GalNAc6S</td><td>4.59
102.5</td><td rowspan="2">3.95
52.2</td><td>3.93
81.0</td><td>4.14
69.0</td><td>3.93
74.0</td><td>4.20
68.3</td><td>NAc: 2.00/23.8</td></tr>
<tr><td>GalNAc</td><td>n.d.</td><td>n.d.</td><td>n.d.</td><td>n.d.</td><td>3.75
62.2</td><td>NAc: 2.03/23.8</td></tr>
</table>

Table S3. ^{1}H (plain) ^{13}C (italic) NMR chemical shift attribution of **CS-11**.

Residue	1	2	3	4	5	6	Other Signals
GlcA3S	4.57<br>105.2	3.58<br>73.7	4.36<br>82.7	3.95<br>78.7	3.79<br>78.3	---	
GalNAc6S	n.d.	3.98<br>52.2	n.d.	4.15<br>68.5	3.87<br>73.5	4.20<br>67.4	NAc: 2.03/23.8

GalNAc	4.52<br>*102.6*		3.79<br>*82.2*	4.09<br>*68.9*	3.63<br>*76.0*	3.77<br>*62.5*	NAc: 2.03/*23.8*

Table S4. ^{1}H (plain) ^{13}C (italic) NMR chemical shift attribution of **CS-12**.

Residue	1	2	3	4	5	6	Other Signals
GlcA2,3S	4.91<br>*102.7*	4.42<br>*79.8*	4.90<br>*78.1*	4.44<br>*78.5*	4.06<br>*79.2*	---	
GalNAc6S	4.67<br>*103.5*	4.01<br>*52.0*	3.83<br>*82.3*	4.22<br>*67.9*	3.93<br>*73.4*	4.22<br>*67.2*	NAc: 2.03/*23.8*
GalNAc	4.62<br>*104.2*			4.17<br>*68.6*	3.70<br>*76.4*	3.79<br>*62.5*	NAc: 2.03/*23.8*

Table S5. Representative Molecular weights of polysaccharides.

Polysaccharide	Mw (kDa)	Mn (kDa)	PI
**CS-1**	70 ± 5	51 ± 4	1.37
**CS-2**	69 ± 5	55 ± 5	1.25
**CS-3**	60 ± 6	51 ± 5	1.17
**CS-4**	45 ± 4	34 ± 3	1.32
**CS-5**	44 ± 5	34 ± 4	1.29
**CS-6**	7.05 ± 0.2	6.7 ± 0.1	1.05
**CS-9**	6.13 ± 0.3	5.9 ± 0.4	1.03
**CS-10**	6.80 ± 0.2	6.53 ± 0.1	1.04
**CS-13**	7.3 ± 0.2	5.57 ± 0.3	1.31
**CS-14**	9.7 ± 0.1	7.40 ± 0.4	1.31
**CS-15**	8.2 ± 0.9	6.12 ± 0.5	1.34

Table S6. Comparison between zeta potential values of several polysaccharides with similar composition.

Polysaccharide	Degree of Sulfation<br>[6S:4S:0S]	Zeta-Potential
**CS-6**	97<br>25:72:3	−16.8 ± 0.265
**[a]**	74<br>25:49:26	−16.6 ± 0.274
**[a]**	50<br>25:25:50	−16.9 ± 0.533
**[a]**	37<br>25:12:63	−17.9 ± 0.865
**[a]**	25<br>25:0:75	−19.8 ± 1.12
**CS-10**	86<br>86:0:14	−21.3 ± 0.917
**CS-9**	75<br>0:75:25	−16.4 ± 0.964

[a] These polysaccharides have been analyzed previously [see: Benito-Arenas, R. et al. *Carbohydr. Polym.* **2018**, *202*, 211-218

3. Supplementary Figures

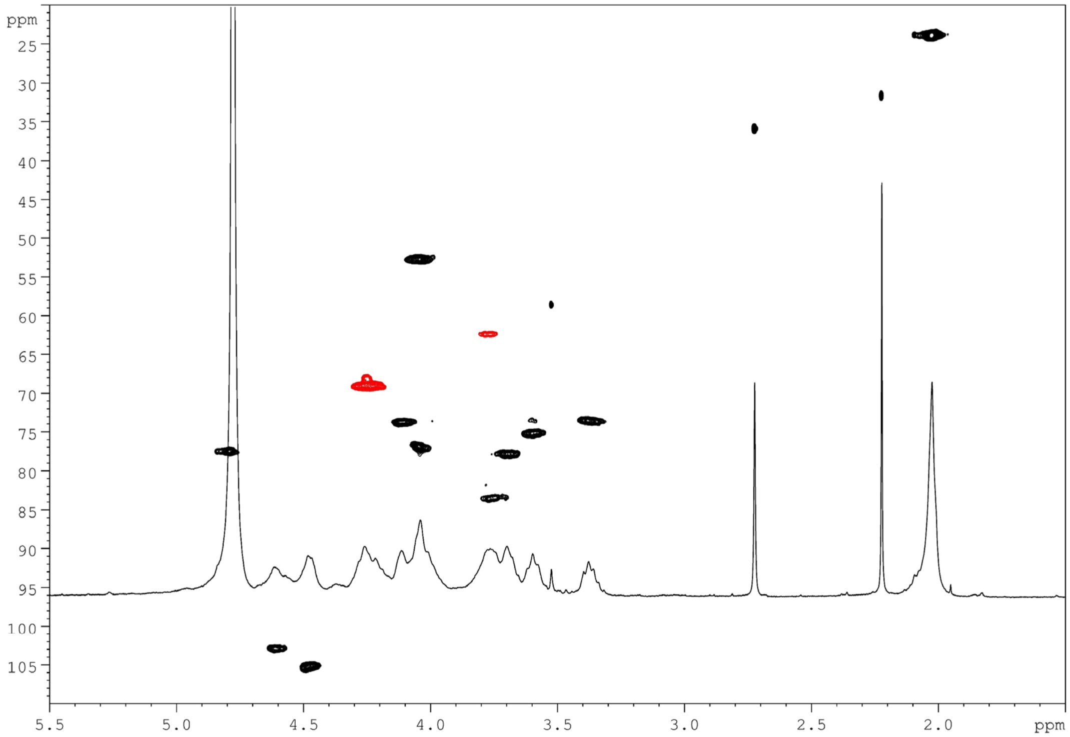

Figure S1. ^{1}H and DEPT-HSQC NMR spectra (400 MHz, D2O, 298 K) of **CS-7.**

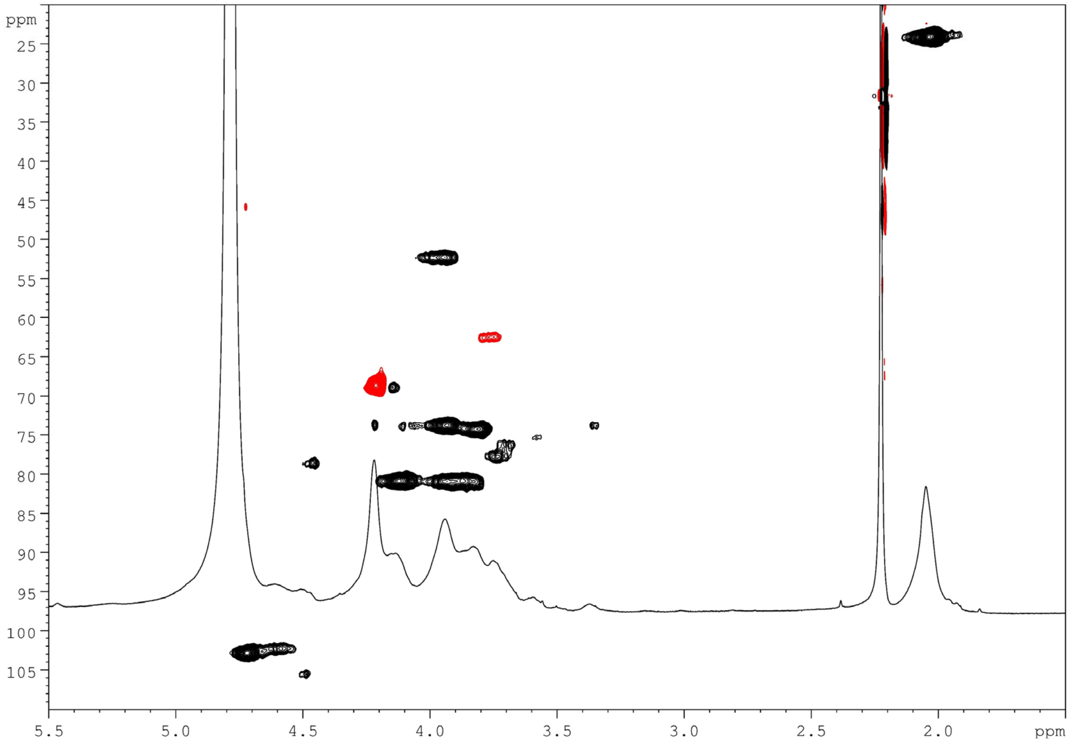

Figure S2. ^{1}H and DEPT-HSQC NMR spectra (400 MHz, D2O, 298 K) of **CS-8.**

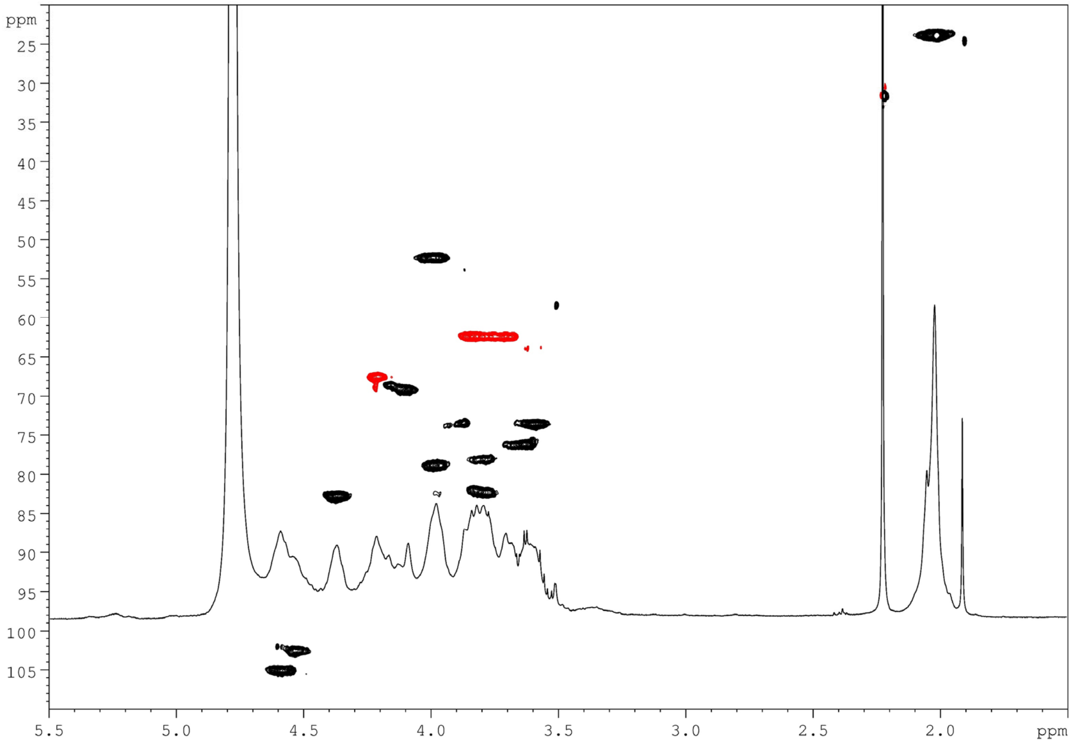

Figure S3. ^{1}H and DEPT-HSQC NMR spectra (400 MHz, D2O, 298 K) of **CS-11.**

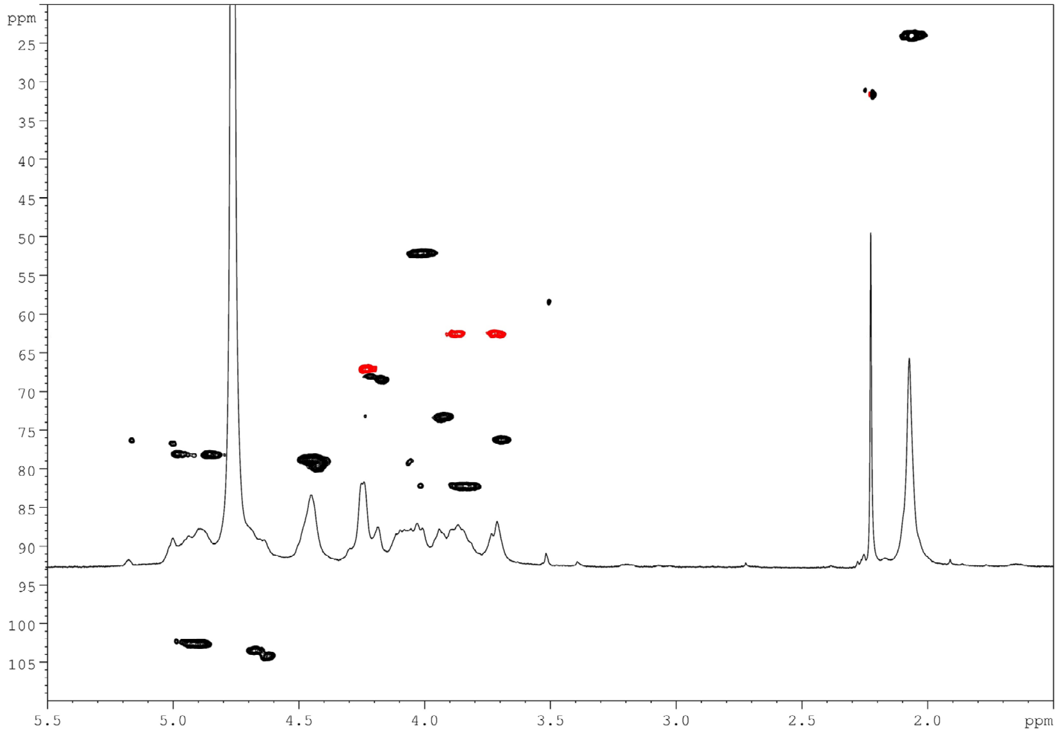

Figure S4. ^{1}H and DEPT-HSQC NMR spectra (400 MHz, D2O, 298 K) of **CS-12**

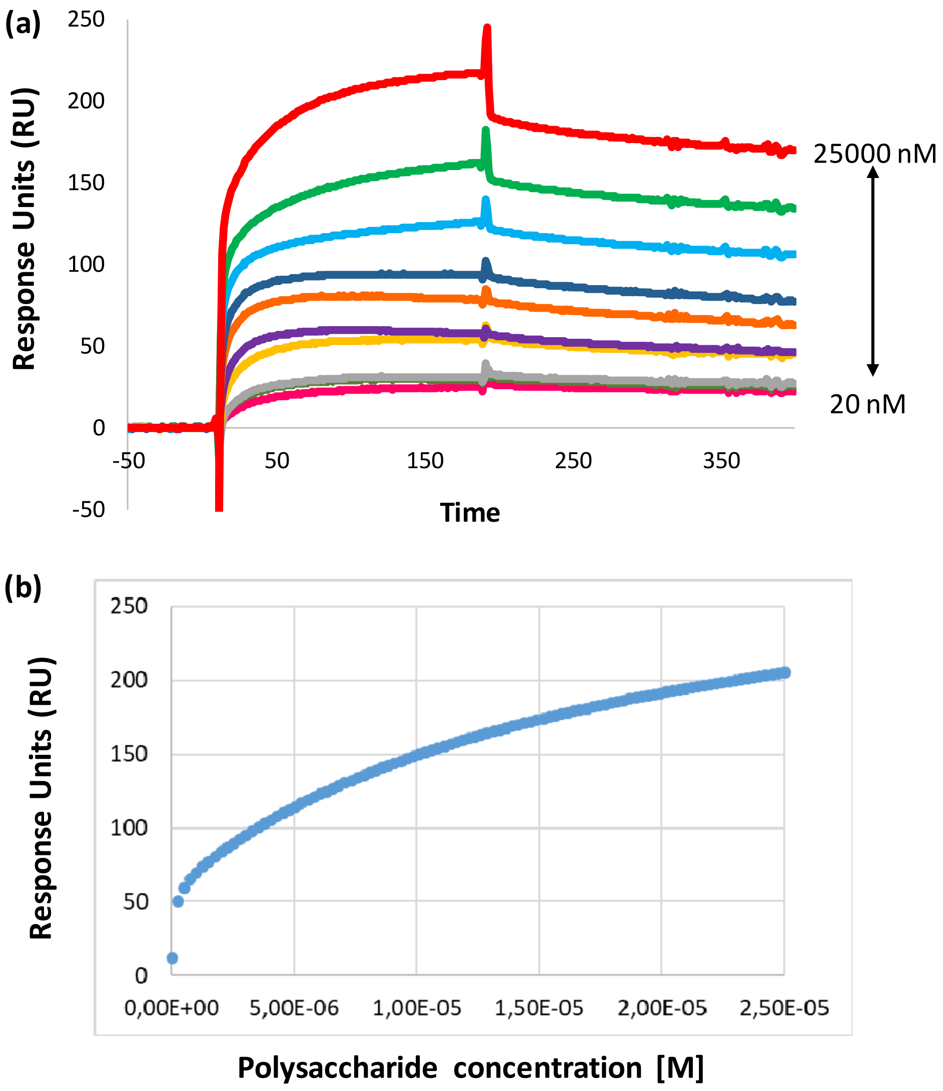

Figure S5. (a) SPR sensograms for binding affinity measurements for FGF-2/**CS-1** interactions. Concentrations of CS-1 (from bottom to top) were: 20 nM, 35 nM, 50 nM, 200 nM, 500 nM, 1000 nM, 2000 nM, 5000 nM, 10000 nM, and 25000 nM. (**b**) Representation of SPR values at the steady state against polysaccharide concentration in the dissociation phase.

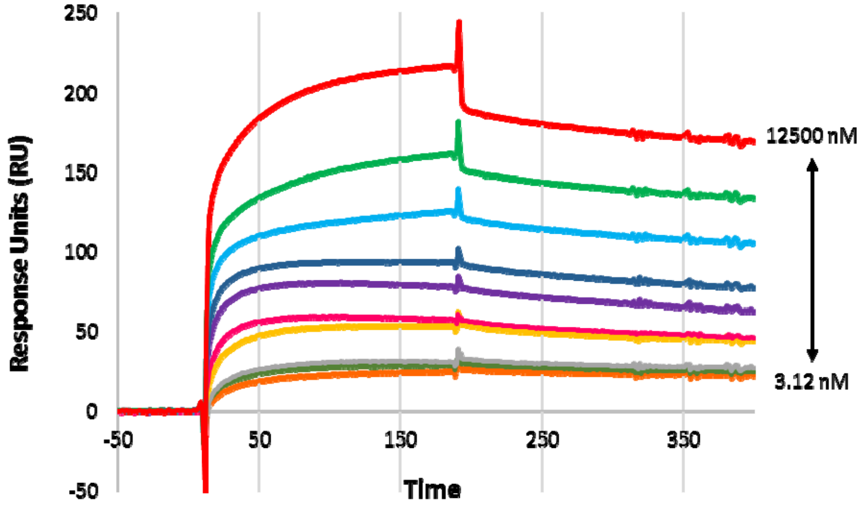

Figure S6. SPR sensograms for binding affinity measurements for FGF-2/**CS-7** interactions. Concentrations of **CS-7** (from bottom to top) were: 3.12 nM, 6.2 nM, 9.37 nM, 12.5 nM, 31 nM, 62 nM, 125 nM, 620 nM, 3120 nM, 12500 nM.

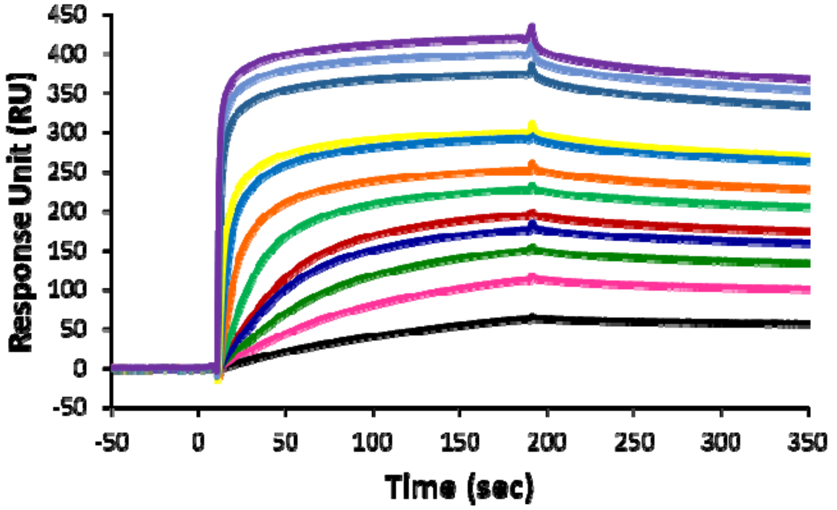

Figure S7. SPR sensograms for binding affinity measurements for FGF-2/**CS-15** interactions. Concentrations of **CS-15** (from bottom to top) were: 10 nM, 20 nM, 35 nM, 50 nM, 75 nM, 100 nM, 200 nM, 500 nM, 1000 nM, 10000 nM, 25000 nM and 51500 nM .

4. References

1. Nagasawa, K.; Inoue, Y.; Tokuyasu, T. An Improved Method for the Preparation of Chondroitin by Solvolytic Desulfation of Chondroitin Sulfates. *J. Biochem*. **1979**, *86*, 1323-1329.

2. Bedini, E.; De Castro, C.; De Rosa, M.; Di Nola, A.; Iadonisi, A.; Restaino, O.F.; Schiraldi, C.; Parrilli, M. A Microbiological-chemical Strategy to Produce Chondroitin Sulfate A,C. *Angew. Chem. Int. Ed.* **2011**, *50*, 6160-6163.
